# Digestive and locomotor capacity show opposing responses to changing food availability in an ambush predatory fish

**DOI:** 10.1242/jeb.173187

**Published:** 2018-06-14

**Authors:** Shi-Jian Fu, Jing Peng, Shaun S. Killen

**Affiliations:** 1 Laboratory of Evolutionary Physiology and Behaviour, College of Life Sciences, Chongqing Normal University, Chongqing 400047, China; 2 Institute of Biodiversity, Animal Health and Comparative Medicine, College of Medical, Veterinary and Life Sciences, University of Glasgow, Glasgow G12 8QQ, UK

**Keywords:** Energy budget, Metabolic rate, Aerobic scope, Teleost fish, Plasticity

## Abstract

Metabolic rates vary widely within species, but little is known about how variation in the ‘floor’ [i.e. standard metabolic rate (SMR) in ectotherms] and ‘ceiling’ [maximum metabolic rate (MMR)] for an individual's aerobic scope (AS) are linked with digestive and locomotor function. Any links among metabolic traits and aspects of physiological performance may also be modulated by fluctuations in food availability. This study followed changes in SMR, MMR, and digestive and locomotor capacity in southern catfish (*Silurus meridionalis*) throughout 15 days of food deprivation and 15 days of refeeding. Individuals downregulated SMR during food deprivation and showed only a 10% body mass decrease during this time. Whereas critical swim speed (*U*_crit_) was robust to food deprivation, digestive function decreased after fasting with a reduced peak oxygen uptake during specific dynamic action (SDA) and prolonged SDA duration. During refeeding, individuals displayed rapid growth and digestive function recovered to pre-fasting levels. However, refed fish showed a lower *U*_crit_ than would be expected for their increased body length and in comparison to measures at the start of the study. Reduced swimming ability may be a consequence of compensatory growth: growth rate was negatively correlated with changes in *U*_crit_ during refeeding. Southern catfish downregulate digestive function to reduce energy expenditure during food deprivation, but regain digestive capacity during refeeding, potentially at the cost of decreased swimming performance. The plasticity of maintenance requirements suggests that SMR is a key fitness trait for in this ambush predator. Shifts in trait correlations with food availability suggest that the potential for correlated selection may depend on context.

## Abstract (precis)

**Summary:** Southern catfish downregulate digestive function and metabolic rate during food deprivation, but regain digestive capacity during refeeding, potentially at the cost of decreased swimming performance.

## INTRODUCTION

Metabolic rate reflects the energetic cost of fueling processes and functions needed to support life (Hulbert and Else, 2000), and is therefore a key physiological trait underlying organismal performance (Biro and Stamps, 2008; Careau and Garland, 2012; Killen et al., 2016b). Beyond the basic energetic requirements to sustain life – termed standard metabolic rate (SMR) in ectotherms (Burton et al., 2011) – an individual can allocate additional energy to other physiological functions such as growth and locomotion. However, an organism can only operate sustainably within the upper bounds set by its aerobic metabolic ceiling, termed maximum metabolic rate (MMR) (Killen et al., 2017; Norin and Clark, 2016). The difference between MMR and SMR is referred to as aerobic scope (AS), and is the capacity for an individual to perform simultaneous oxygen-consuming physiological tasks (Clark et al., 2013; Fry, 1971). SMR, MMR and AS vary greatly both among and within species. Although this variation is generally repeatable among individuals (Killen et al., 2016a; Norin and Malte, 2011, 2012), metabolic traits also show plasticity within individuals across different environmental contexts (Fu et al., 2005; Norin et al., 2016).

To date, however, the links between SMR, MMR and AS traits at the individual level and their relationships with other dimensions of performance capacity remain unclear. For example, having a higher SMR may limit the capacity for locomotor and growth performance because it is energetically expensive and may also reduce an individual's aerobic scope (as predicted by the allocation model; Careau et al., 2008). In contrast, however, a high SMR could reflect the maintenance costs of greater ‘metabolic machinery’ that facilitates a higher metabolic ceiling and aerobic scope for accommodating additional physiological functions, including digestion and physical activity (production model; Auer et al., 2017; Careau et al., 2008; Killen et al., 2010, 2016b). In ectotherms in particular, the additional metabolic cost of digestion and nutrient assimilation – termed specific dynamic action (SDA) – can be substantial and occupy a large proportion of an individual's AS (Norin and Clark, 2017; Secor, 2009). Individuals with a high SMR appear to possess increased food processing capacity and can thus consume more food when resource availability is high (Auer et al., 2015; Millidine et al., 2009). However, the additional role of the MMR in modulating this capacity has not been examined. For example, individuals with a higher MMR may be less physiologically constrained during digestion, or may be able to process meals faster (by diverting more aerobic capacity to digestion), leading to a reduced duration of SDA and period of potentially constrained locomotor ability (Norin and Clark, 2017).

The possible effects of SMR and MMR on performance capacity are further complicated by the plasticity of metabolic traits in response to changing environmental factors (Killen et al., 2013; Van Leeuwen et al., 2012). Fluctuations in food intake, for example, are commonly experienced by animal species (McCue, 2010; Wang et al., 2006). Food availability in nature can change drastically in response to shifting abiotic conditions (e.g. changes in temperature or oxygenation; Biro et al., 2004; Post and Parkinson, 2001). In addition, predation threat can limit feeding opportunities for prey species, which can lead to diminished growth or condition (Killen et al., 2007; Killen and Brown, 2006). For many fish species it is not uncommon for individuals to survive days, weeks or months without eating during periods of overwintering or eutrophication during the summer (Biro et al., 2004; Hervant et al., 2001). Extended periods of starvation in fish can be accompanied by a reduction in SMR (O'Connor et al., 2000; Pang et al., 2016; Yang and Somero, 1993), possibly as an adaptive response to reduce energy expenditure, although effects on other metabolic traits are less well known. Locomotor ability is also reduced by long-term food deprivation (Killen et al., 2014; Martínez et al., 2004, 2002), presumably due to protein degradation and reduced function of aerobic and anaerobic enzymes in muscle tissue. It is also possible that there may be a direct reduction in MMR and AS during fasting, which would further constrain locomotor ability during food deprivation. Even after refeeding following a period of food deprivation, reduced locomotor performance can persist, possibly due to the negative effects of compensatory growth (Metcalfe and Monaghan, 2001). A possibility that has not been thoroughly investigated is that periods of food deprivation and refeeding may disrupt correlations among metabolic traits and performance capacity at the individual level, or alter trait repeatability (Killen et al., 2016a). These effects could affect the degree to which traits can be targets for direct or correlated selection in response to evolutionary pressures (Kern et al., 2016; Lande and Arnold, 1983).

List of symbols and abbreviations

AS: aerobic scope
FAS: factorial aerobic scope
GLM: general linear model
LME: linear mixed effects model
MMR: maximum metabolic rate
PMR: peak metabolic rate after feeding
PMS: peak metabolic scope
SDA: specific dynamic action
SMR: standard metabolic date

We examined these issues in southern catfish (*Silurus meridionalis* Chen 1977), an ambush predator common in the Yangtze and Zhujiang rivers, where it experiences wide seasonal fluctuations in food availability. We estimated metabolic rate at rest, during swimming and after feeding via rate of oxygen uptake, which, although not accounting for ATP supplied by various anaerobic metabolic pathways, is a common proxy for aerobic metabolic rate in animals (Chabot et al., 2016). First, we measured the effect of 15 days of food deprivation followed by 15 days of refeeding on metabolic rates, AS and feeding and digestive costs. It was hypothesized that food deprivation would cause a decrease in SMR and MMR due to plastic downregulation of maintenance metabolism and maximum aerobic capacity due to changing prioritization of energy allocation and possible catabolism of skeletal muscle tissue, but that these variables would show recovery after refeeding. Similarly, it was anticipated that digestive function (as estimated by SDA) and swimming capacity would also show decreases and recovery in response to food deprivation and refeeding. We also investigated how relationships among metabolic traits (e.g. SMR, MMR and AS) and measures of digestive and locomotor capacity vary among individuals and across the different feeding periods. It was hypothesized that plastic variation in energy allocation to various functions in response to changing conditions of food availability would alter correlations among suites of traits related to organismal performance.

## MATERIALS AND METHODS

### Experimental animals

This study was approved by the Animal Care and Use Committee of Key Laboratory of Animal Biology of Chongqing, China (permit number: Zhao-20141015-01) and performed in strict accordance with the recommendations in the Guide for the Care and Use of Animals at the Key Laboratory of Animal Biology of Chongqing, China.

Juvenile southern catfish of unknown sex were obtained from a local hatchery (Hechuan, Chongqing, China) on the Yangtze River. The fish were transported to Chongqing Normal University and maintained in an indoor re-circulating rearing system for 4 weeks. During this period, the temperature of the fresh dechlorinated water was maintained at 25±1°C. The photoperiod was 14 h: 10 h light:dark, and oxygen tension was maintained above 80% saturation oxygen tension. One tenth of the water was replaced daily. The fish were fed once daily to satiation at 09:00 h with cutlets of freshly killed loach (*Misgurnus anguillicaudatus*). Any remaining food and feces were removed 1 h after feedings.

### Experimental protocol

The overall strategy was to measure fish for metabolic traits, and indices of digestive and locomotor capacity during three experimental periods: (1) initial measures; (2) after 15 days of food deprivation; and (3) after a 15 day period of refeeding. This design used repeated measures whereby every fish (*n*=40; wet mass=4.95±0.09 g; total length=8.18±0.06, mean±s.e.m.) in the study was measured for all variables during each experimental period (with the exception of two fish that died of unknown causes before being measured for variables after the refeeding period). After 4 weeks of laboratory acclimation in the re-circulating water system, experimental fish were transferred individually into a Blazka swimming chamber for measurement of the critical swimming speed and oxygen uptake during swimming. The fish were then transferred to individual flow-through respirometers for measurement of oxygen uptake before (i.e. to estimate SMR) and after (i.e. specific dynamic action, SDA) feeding on a meal of freshly killed loach consisting of 10% of each fish's body mass. Fish remained in chambers for 15 days without feeding and then the *U*_crit_ and SDA were measured again using the same procedure as at the beginning of the study. Then, while being held individually in respirometers, fish were fed to satiation each day with freshly killed loach for another 15 days, after which SMR, SDA and swimming performance were measured again.

### Measurement of variables

#### Measurement of swimming performance

A Blazka-type swimming tunnel respirometer with a 20 cm^2^ cross-sectional area swim chamber was used to measure the *U*_crit_ of the fish (total volume 3.5 l). After being fasted for at least 36 h and measured for morphological measurements, the fish were individually transferred into the swim chamber and held for 4 h at a 3 cm s^−1^ water velocity. This speed elicits minimal movement but provides mixing of water and oxygenation within the flume. The flow of aerated water through the respirometer was maintained continuously during this recovery period. The water temperature in the swim chamber was controlled to 25±0.2°C using a water bath connected to a thermostat and stainless steel heat exchanger. After the acclimation period, water velocity was adjusted to 8 cm s^−1^ and then increased by 8 cm s^−1^ increments every 20 min until the fish fatigued. Fatigue was defined as the failure of the fish to move away from the rear honeycomb screen of the swimming chamber for 20 s (Lee et al., 2003). The *U*_crit_ was calculated for each individual fish using Brett's equation (Brett, 1965):(1)
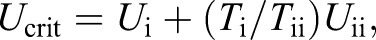


where *U*_i_ is the highest speed at which the fish swam a complete 20 min duration during the trial (cm s^−1^), *U*_ii_ is the velocity increment (8 cm s^−1^), *T*_ii_ is the duration of a complete stepwise speed increment (20 min) and *T*_i_ is the time that the fish swam at the final speed (min). To adjust for the solid blocking effect described by Bell and Terhune (Bell and Terhune, 1970), the calculated *U*_crit_ of each fish was corrected using the following equation (Jain et al., 1998):(2)
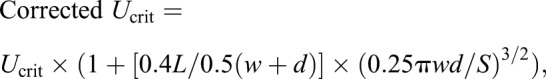


where *L* is body length (cm), *w* is the maximal body width (cm), *d* is the maximal body depth (cm) and *S* is the internal cross-sectional area of swimming tunnel (cm^2^). To measure oxygen uptake during swimming, a small fraction of the water from the sealed respirometer was siphoned past the probes of an oximeter (HQ30, Hach Company, Loveland, CO, USA) in a cuvette that was thermoregulated with a water bath. When open, respirometers were supplied with thermoregulated water that circulated in a reservoir tank at a flow rate of 500 ml min^−1^. When closed, the oxygen concentration (mg l^−1^) in the water was recorded once every 2 min. Fish were removed from the flume after fatigue and bacterial oxygen consumption was measured. The oxygen uptake (*Ṁ*_O_2__; mg O_2_ h^−1^) of an individual fish during swimming was calculated based on the depletion of oxygen according to the following equation:(3)
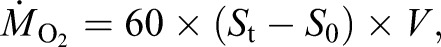


where *S*_t_ and *S*_0_ (mg l^−1^ min^−1^) represent the decrease in the water oxygen concentration per minute with and without fish (bacterial metabolism), respectively. These values were obtained from linear regressions of time (min) and water oxygen concentration (mg l^−1^). *V* is the total volume of the respirometer (3.1 l) minus the volume of the fish. The water oxygen content in the respirometer was never allowed to fall below 85% oxygen saturation (Claireaux et al., 2006). The maximum *Ṁ*_O_2__ during swimming was used as the value for MMR during the *U*_crit_ test (only *T*_i_>10 min was considered in the analysis when the fish swam at the fastest speed). Despite being a benthic predator, *S. meridionalis* swim well in flumes and are not resistant to swimming against a current. In the wild, this species lives in rivers, commonly swimming against currents when moving to spawning grounds.

#### Estimation of SMR and post-feeding oxygen uptake

The post-feeding *Ṁ*_O_2__ of individual fish was measured using a continuous-flow respirometer. Forty fish were placed in individual continuous-flow respirometer chambers after at least 36 h of fasting and were allowed to acclimate to the respirometers for another 12 h. The *Ṁ*_O_2__ was measured ten times in 2-h intervals before feeding. The mean of the lowest three values out of the ten measures was used as an estimate of SMR and considered the baseline level of oxygen uptake before feeding. The respirometers were custom designed with two additional ports: one for the addition of food and one for allowing for the collection of waste material. After the measurement of SMR, food was added into respirometers through the feeding port. All food was always eaten within a few minutes. The *Ṁ*_O_2__ was measured at 2-h intervals for 48 h, which ensured that all fed fish completed their digestion. The following formula was used to calculate the *Ṁ*_O_2__ (*X*_m_; mg O_2_ h^−1^):(4)
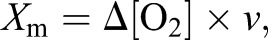


in which Δ[O_2_] is the difference (mg O_2_ l^−1^) in oxygen concentrations between the experimental chamber and the control chamber (chamber without fish), and *v* is the water flow rate in the chamber (l h^−1^). The dissolved oxygen concentration was measured at the outlet of the chamber with an oximeter (HQ30d, Hach Company). The flow rate of water through the respirometer chamber was measured by collecting the water outflow from each chamber into a beaker over 2 min. The flow rate in each chamber was adjusted to assure at least 70% saturation of the dissolved oxygen concentration in the outlet water to avoid undue stress. Light was maintained during the entire experimental period to minimize the effect of circadian rhythms on *Ṁ*_O_2__ (Fu et al., 2006). Both sides of the chamber were covered by opaque plastic to avoid the visual contact between neighboring individuals. The total increase in oxygen consumption above baseline was defined as SDA and was calculated as the area under the curve from the onset of feeding and until the time post-feeding when the *Ṁ*_O_2__ was not significantly different from the pre-fed level (SDA duration). The peak of *Ṁ*_O_2__ (PMR) was defined as the observed maximum O_2_ uptake rate in the SDA process. The available AS during peak *Ṁ*_O_2__ after feeding [peak metabolic scope (PMS)] was estimated as the difference between MMR and PMR. One chamber without a fish acted as a control for background O_2_ consumption. Any feces produced in chambers would accumulate at the exit port and this would be opened to release the waste and prevent bacterial accumulation. Chambers were also cleaned between feeding periods. The blank chamber and treatment chambers were being exposed to the same source water and so did not differ in the level of bacterial respiration.

#### Body size and growth performance

Body mass and total body length were measured after each experimental period. Rates of mass loss or growth were estimated in terms of body mass and total length, and were calculated between measurement periods according to the equation:(5)
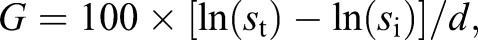


where *s*_t_ is the body mass or standard length at time *t*, *s*_i_ is the initial body mass or standard length and *d* is the time elapsed in days (Hopkins, 1992).

### Data analysis

All models were produced using R v. 3.4.0 (http://www.R-project.org/) using the function lmer in package lme4 (Bates et al., 2016). Initial linear mixed effects models (LMEs) were constructed to examine the effects of food deprivation and refeeding on fish size, with either body mass or total length as the response variables, and feeding period (initial, fasted, refed) as a categorical explanatory variable, and fish ID as a random effect. The effects of feeding period on metabolic traits were examined with LMEs using either SMR, MMR, AAS or FAS as response variables, body mass as a continuous explanatory variable (to control for variation in fish size among and within feeding periods), feeding period as a categorical response variable, and fish ID as a random effect. Factors influencing measures associated with swimming and post-prandial metabolism were examined with LMEs using either *U*_crit_, SDA duration, PMR or PMS as response variables, SMR, MMR and body mass as continuous explanatory variables, feeding period as a categorical response variable, and fish ID as a random factor. Two-way interactions between feeding period and other factors were initially included in all models but removed when non-significant, and the models re-run. Model assumptions of linearity, normality and homogeneity of residuals were verified by inspecting plots of model residuals versus fitted values. When necessary, body mass, SMR, MMR, AAS, PMR, PMS and SDA were log transformed. For visual representation in figures, values for all response variables are adjusted to that of a 9 g animal, the mean mass of all fish over all feeding periods. This was achieved by adding the residual value of each response variable (logged when necessary to provide a linear relationship; Fig. S1) versus log body mass (g) (or total length in the case of *U*_crit_) to the fitted value for each linear relationship at 9 g.

Significance testing was employed to provide some indication of the strength of evidence for observed patterns, along with model *r*^2^ values using the MuMIn 1.9.13 package for R (Bartoń, 2013). This included marginal *r*^2^ (*r*^2^_m_) and conditional *r*^2^ (*r*^2^_c_), which indicate the variance explained by fixed factors, and by both fixed and random factors, respectively (Nakagawa and Schielzeth, 2013). *P*-values are generally imprecise in model outputs and are arbitrary when used as thresholds for declaring statistical significance, and are problematic and limiting in several ways (Boos and Stefanski, 2011; Halsey et al., 2015). Thus, for all models, we treat *P*-values as a continuous measure providing an approximate level of evidence against the null hypothesis (Fisher, 1956).

Across-context repeatability of individual SMR, MMR, PMR, PMS, *U*_crit_ and SDA duration were calculated as adjusted (consistency) repeatability according to Nakagawa and Schielzeth (2010), using variances calculated with LMEs that included feeding period and mass as fixed effects and fish ID as a random effect.

## RESULTS

### Effect of fasting and refeeding on metabolic traits, swimming ability and digestive capacity

**Fig. 1.**
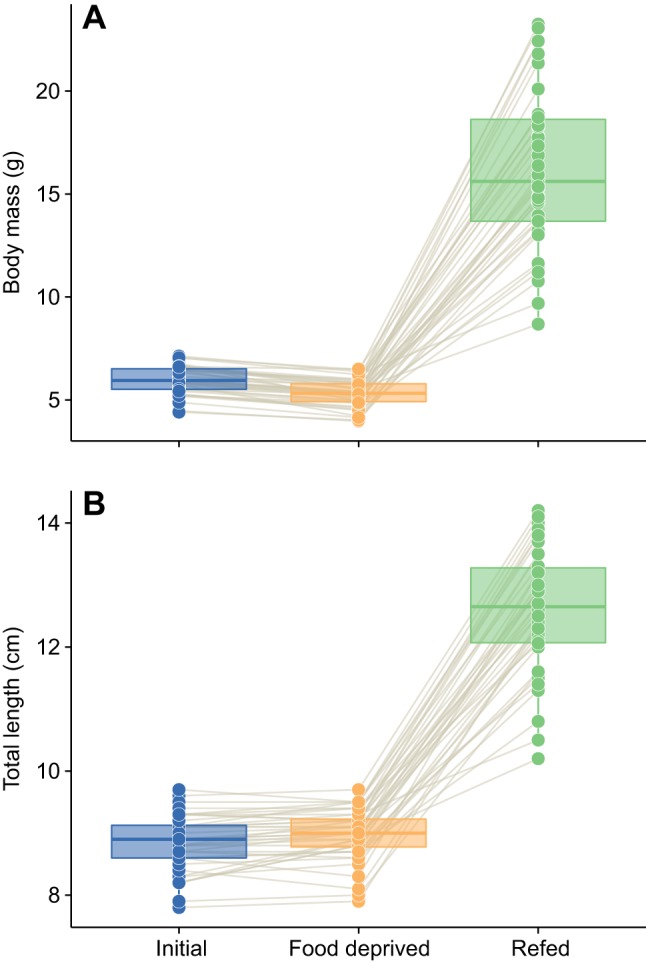
**Changes in body mass and total length for individual southern catfish under conditions of normal feeding, after 15 days of food deprivation, and 15 days after resuming normal feeding.** (A) Body mass. (B) Total length. Each fish (*n*=40, except in the refed condition where *n*=38) was measured under each scenario; the thin brown lines connect data points for each individual in each feeding period. Boxplot upper and lower hinges represent the 25th and 75th percentiles, respectively; the horizontal line within the box represents the median; the length of whiskers represents the range of data points between each hinge and 1.5× the difference between the 25th and 75th percentiles. Data beyond these limits are outliers. See Table S1 for results of statistical models.

After 15 days of food deprivation, the body mass of fish had declined by only 10% on average and was not significantly different from initial values (Fig. 1; Table S1). Body length showed no change during this time (Table S1). During 15 days of refeeding, fish grew quickly: mass increased by approximately 200% (Fig. 1; Table S1; LME, effect of feeding period, *t*=21.85, *P*<0.0001) and length increased by about 36% on average during this time (LME, effect of feeding period, *t*=31.19, *P*<0.0001).

**Fig. 2.**
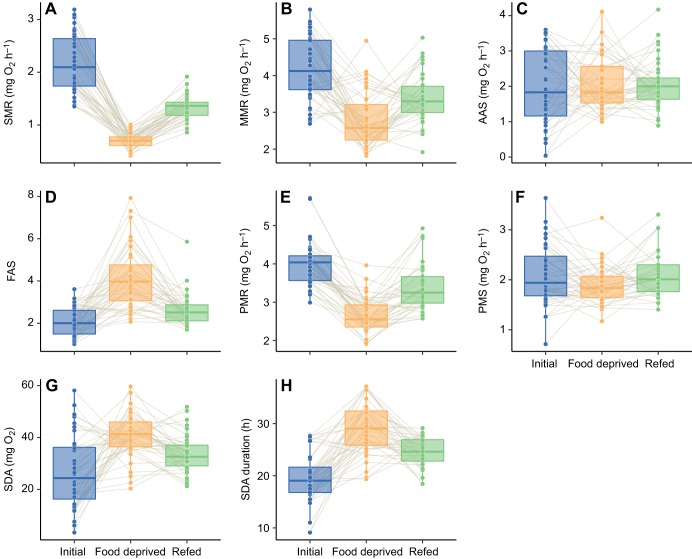
**Changes in metabolic traits and indices of digestive and locomotor performance for individual southern catfish under conditions of normal feeding, after 15 days of food deprivation and 15 days after resuming normal feeding.** (A) Standard metabolic rate (SMR). (B) Maximum metabolic rate (MMR). (C) Absolute aerobic scope (AAS). (D) Factorial aerobic scope (FAS). (E) Peak metabolic rate (PMR). (F) Peak metabolic scope (PMS). (G) Specific dynamic action (SDA). (H) SDA duration. Each fish (*n*=40, except refed condition where *n*=38) was measured under each scenario; the thin brown lines connect data points for each individual in each feeding period. Rates of oxygen uptake and SDA are adjusted to a common body mass of 9 g (the mean mass of all individuals during measurement across the duration of the study). Boxplot upper and lower hinges represent the 25th and 75th percentiles, respectively; the horizontal line within the box represents the median; the length of whiskers represents the range data points between each hinge and 1.5× the difference between the 25th and 75th percentiles. Data beyond these limits are outliers. See Tables S1 and S2 for results of statistical models.

After adjusting for differences in body mass among feeding periods, SMR and MMR decreased with fasting (Fig. 2A,B; Table S1; SMR: LME, effect of feeding period, *t*=−20.79, *P*<0.0001; MMR: LME, effect of feeding period, *t*=−5.73, *P*<0.0001), then began to approach initial levels with refeeding. Initial analyses suggested that mass-adjusted AAS did not change with fasting or refeeding, whereas FAS increased during fasting (Fig. 2C,D; Table S1). However, subsequent models that included effects of SMR on AAS revealed that, after food deprivation, fish with a higher SMR actually had a lower AAS when compared to initial values (Table S2; LME, effect of SMR, *t*=−3.45, *P*<0.0001). SMR and AAS both showed moderate across-context repeatability, whereas MMR and FAS showed no evidence of repeatability across feeding periods (Table 1).

**Table 1.**
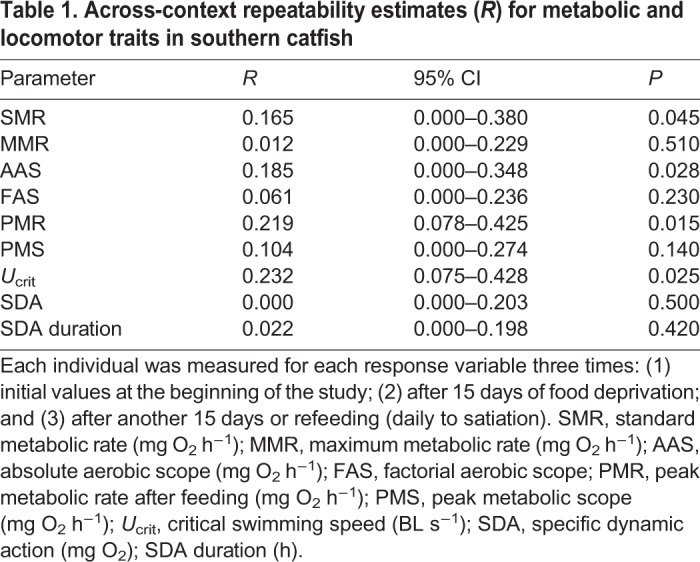
**Across-context repeatability estimates (*R*) for metabolic and locomotor traits in southern catfish**

**Fig. 3.**
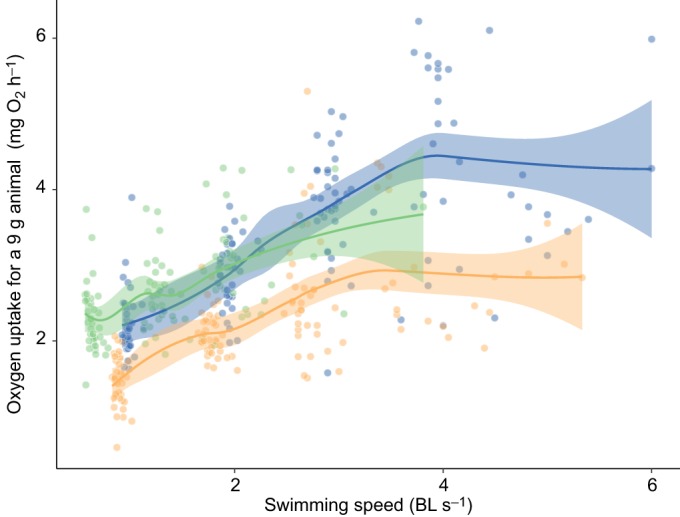
**Changes in oxygen uptake with swimming speed of southern catfish.** Data were obtained under conditions of normal feeding (blue), after 15 days of food deprivation (orange) and 15 days after resuming normal feeding (green). Each fish was measured under each scenario; each data point is data for an individual at a given speed. Rates of oxygen uptake are adjusted to a common body mass of 9 g (the mean mass of all individuals during measurement across the duration of the study). For illustrative purposes, solid lines represent LOESS curves; see Table S3 for parameters and results of statistical models. See Fig. S2 for plot using swimming speed expressed in absolute terms (cm s^−1^). Shaded areas around lines represent the 95% confidence intervals. BL, body lengths.

**Fig. 4.**
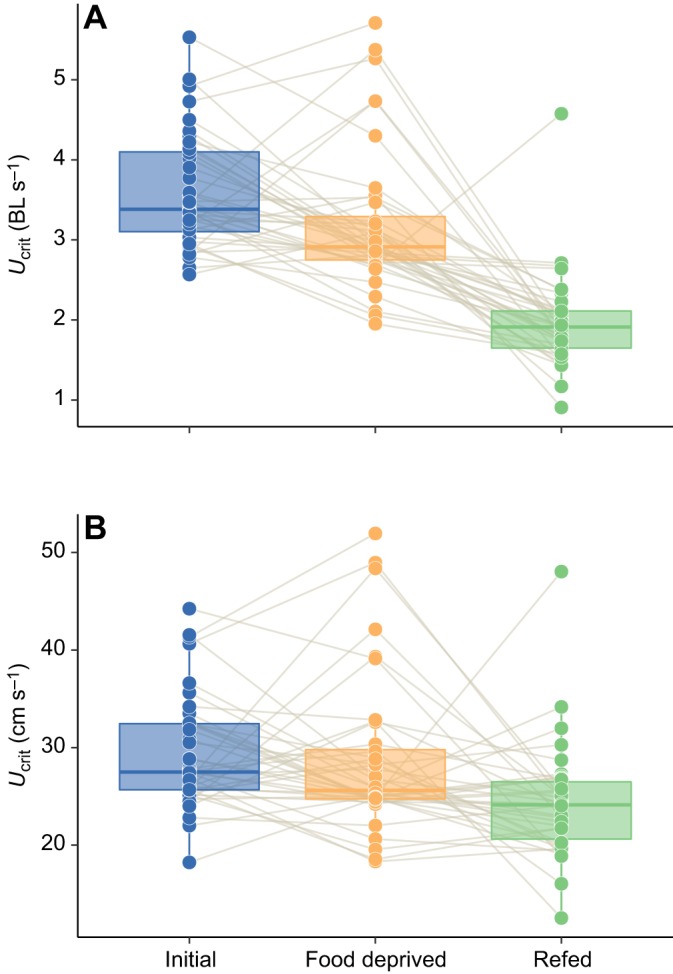
**Changes in critical swimming speed (*U*_crit_) for individual southern catfish under conditions of normal feeding, after 15 days of food deprivation and 15 days after of resuming normal feeding.** Data are expressed in (A) relative terms [body length (BL) s^−1^] and (B) absolute terms (cm s^−1^). Each fish (*n*=40, except refed condition where *n*=38) was measured under each scenario; the thin brown lines connect data points for each individual in each feeding period. Boxplot upper and lower hinges represent the 25th and 75th percentiles, respectively; the horizontal line within the box represents the median; the length of whiskers represents the range data points between each hinge and 1.5× the difference between the 25th and 75th percentiles. Data beyond these limits are outliers. See Table S4 for results of statistical models.

**Fig. 5.**
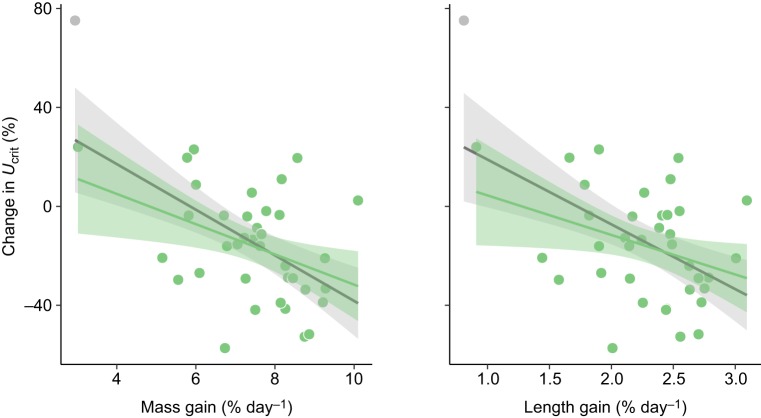
**Relationships between the change in critical swimming speed (*U*_crit_) for southern catfish before and after 15 days of food deprivation followed by 15 days of refeeding, and growth rate during refeeding.** Each data point represents one individual (*n*=40, except refed condition where *n*=38). The gray point represents an outlying data point. For illustrative purposes, solid lines represent linear regressions; see Table S5 for parameters and results of statistical models. The gray line represents the relationship with the outlier included; the green line represents data with the outlier removed. Shaded areas around lines represent the 95% confidence intervals.

Oxygen uptake increased with swimming speed in all three feeding periods but reached a plateau in the initial and food-deprived fish, likely due to anaerobic metabolism being a major source of energy supply with further increases in speed (Fig. 3, Fig. S2; Table S3). Across swimming speeds, oxygen uptake during swimming decreased during food deprivation, but this trend was not significant (Table S3). Although *U*_crit_ showed no significant change from initial values in response to fasting (Fig. 2G; Table S4), there is evidence that swimming performance was decreased compared to initial values after refeeding. Specifically, after refeeding, fish displayed a decreased absolute *U*_crit_ compared with initial values, despite having reached a greater total body length after refeeding, which would normally be expected to increase absolute *U*_crit_ (Fig. 4; Table S4). In addition, the change in *U*_crit_ observed for individuals across feeding periods was negatively correlated with specific growth rate in terms of both mass gain [general linear model (GLM), *t*=−3.684, *P*=0.0008] and body length gain (GLM, *t*=−3.176, *P*=0.003) during the refeeding period (Fig. 5; Table S5). Trends remained consistent when analyses were repeated with the exclusion of a potential outlier (gray point, Fig. 5, Table S5). *U*_crit_ showed moderate repeatability across feeding periods (Table 1). Mass loss and growth rate, in terms of mass or length, were not related to either SMR or AS during either the food-deprivation or refeeding periods (Fig. S3).

**Fig. 6.**
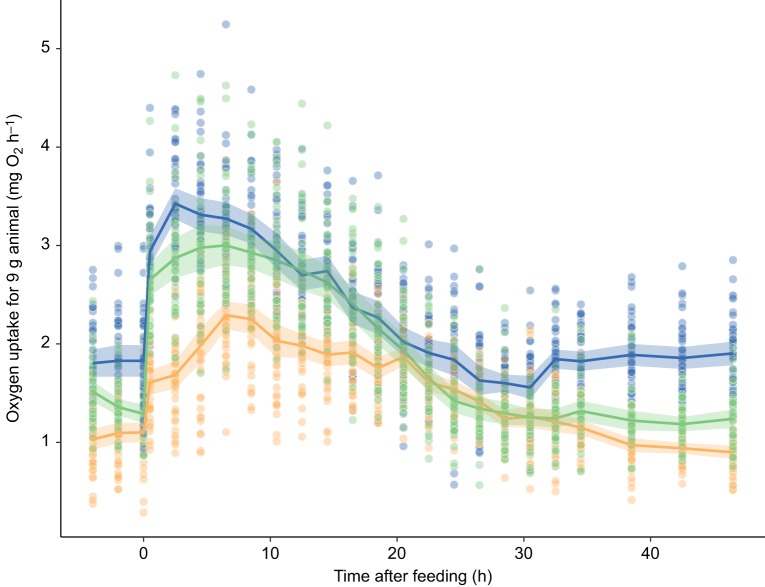
**The post-feeding changes in oxygen uptake of southern catfish.** Data were obtained under conditions of normal feeding (blue), after 15 days of food deprivation (orange) and 15 days after resuming normal feeding (green). Each fish (*n*=40, except refed condition where *n*=38) was measured under each scenario; each data point is data for an individual at a given time point. Rates of oxygen uptake are adjusted to a common body mass of 9 g (the mean mass of all individuals during measurement across the duration of the study). For illustrative purposes, solid lines are connections between mean of points at each time interval within each feeding treatment; see Table S2 for results of statistical models. Shaded areas around lines represent the 95% confidence intervals.

Oxygen uptake quickly increased after food intake until reaching a peak value (PMR), then gradually decreased to baseline values. However, the shape of this profile varied among feeding periods (Fig. 6) and was modulated by interactions with individual SMR (Table S2). PMR decreased after fasting and was still reduced below initial levels after 15 days of refeeding (Fig. 2E; LME, effect of feeding period, *t*=5.12, *P*<0.0001). Results for PMS were similarly complex: PMS showed no change across feeding periods (Fig. 2F) but, after accounting for variation in SMR, fasted and refed fish both had higher PMS values compared with initial values (Table S2; LME, SMR×feeding period interaction, *P*<0.0001). SDA duration increased by approximately 50% after fasting but returned to initial levels after refeeding (Fig. 2H; Fig. 3; Table S2; LME, effect of feeding period, *t*=5.77, *P*<0.0001). Body mass had a significant effect on all indices of digestive performance except SDA duration (Fig. S1; Table S2). PMR showed moderate repeatability across feeding periods, whereas PMS and SDA duration showed low repeatability (Table 1).

### Effects of feeding period on links among traits

**Fig. 7.**
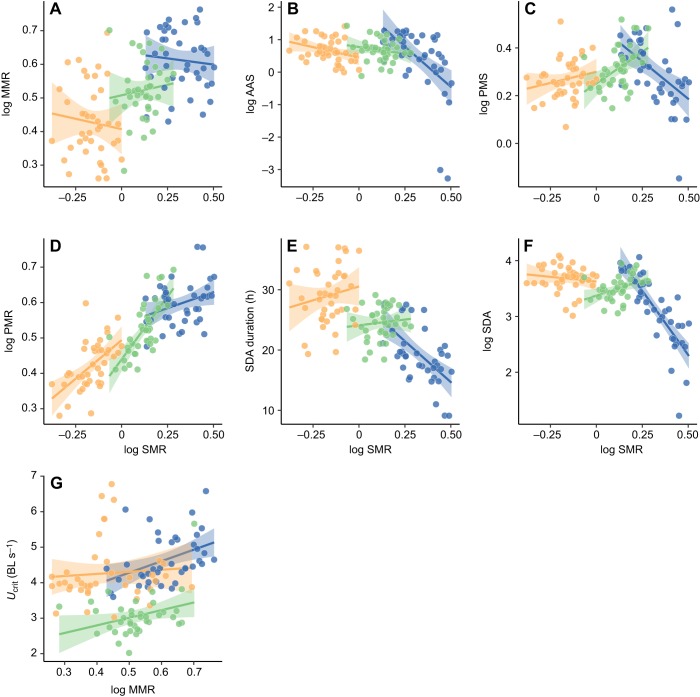
**Correlations among metabolic** **traits and indices of digestive and locomotor function for southern catfish.** (A) MMR (mg O_2_ h^–1^). (B) AAS (mg O_2_ h^–1^). (C) PMS (mg O_2_ h^–1^). (D) PMR (mg O_2_ h^–1^). (E) SDA duration. (F) SDA (mg O_2_). (G) *U*_crit_. The *x*-axes show SMR (mg O_2_ h^–1^) (A-F) and MMR (mg O_2_ h^–1^) (G). Data were obtained under conditions of normal feeding (blue), after 15 days of food deprivation (orange) and 15 days after resuming normal feeding (green). Each fish (*n*=40, except refed condition where *n*=38) was measured under each scenario; each data point is data for one individual. Rates of oxygen uptake are adjusted to a common body mass of 9 g (the mean mass of all individuals during measurement across the duration of the study). For illustrative purposes, solid lines represent linear regressions; see Table S2 for parameters and results of statistical models. Shaded areas around lines represent the 95% confidence intervals.

Overall, there was little evidence of a correlation between SMR and MMR (Fig. 7A; Table S2). There was a negative association between SMR and AAS among individuals regardless of feeding period (Fig. 7B, Table S2; LME, effect of SMR, *t*=−3.45, *P*<0.001). SMR showed a positive correlation with PMR, particularly in the fasted and refed periods (Fig. 7D, Table S2; LME, SMR×feeding period interaction, *P*<0.0001). Although PMS was negatively correlated with SMR initially, it was positively correlated with SMR in the fish after fasting and refeeding (Fig. 7C, Table S2; LME, SMR×feeding period, *P*<0.0001). Overall, fish with a higher SMR had a shorter SDA duration (Fig. 7E, Table S2; LME, effect of SMR, *t*=−5.14, *P*<0.0001). However, the exact nature of this correlation across individuals varied depending on the feeding period. Specifically, SMR showed a strong negative correlation with SDA duration initially but then showed a positive correlation after fasting. In other words, although fasted fish had an overall decreased SMR with correspondingly high SDA durations (compared to the dataset as a whole), within the fasting period the opposite trend was observed in that fish with a higher SMR actually showed a longer SDA duration (LME, SMR×feeding period interaction, *P*<0.0001). MMR was positively linked with *U*_crit_ across all feeding periods (Fig. 7G; Table S4).

## DISCUSSION

These results reveal a number of complex relationships among metabolic traits and aspects of organismal performance related to locomotor and digestive capacity under conditions of changing food availability. Notably, locomotor and digestive responses to changing availability were in opposition: swimming ability was robust to food deprivation but appeared negatively affected during refeeding, whereas digestive ability was depressed during food deprivation but increased rapidly upon refeeding. In conjunction with a strong downregulation in SMR during food deprivation, these effects also altered correlations among traits across feeding regimes.

### Fasting reduced metabolic rate, digestive capacity and locomotor ability

Initial estimates of SMR in the present study were within the range of previous studies in southern catfish (Fu et al., 2005), but much lower than those of other fish species measured under similar conditions (Fu et al., 2009). This low SMR plus the downregulation of metabolism during fasting likely explains why more than 2 weeks of food deprivation only resulted in, on average, a 10% decrease in body mass. Similar downregulation of SMR has been noted in other species, possibly as an adaptation to reduce energy expenditure during food deprivation, but generally after a longer time period without food (O'Connor et al., 2000; Yang and Somero, 1993). For example, black carp (*Mylopharyngodon piceus*) only show a 30% decrease in SMR after 21 days of fasting at 25°C, whereas, in the present study, southern catfish showed a 60% decrease in SMR after 2 weeks (Pang et al., 2016). In the current study, it is possible that changes in spontaneous activity within the respirometers may have contributed to the observed differences in estimated SMR among feeding periods. However, southern catfish are inactive in respirometers, performing whole-body movements extremely infrequently (Fu et al., 2009). The results of the current study indicate that southern catfish may be more physiologically responsive to short-term fluctuations in food availability, possibly as an adaptation to tolerating variable food availability.

The effects of food deprivation varied profoundly between indices of digestive and locomotor ability. Although MMR decreased with fasting, AS and *U*_crit_ appeared robust, showing no change from initial values in response to food deprivation. Indeed, fasted fish used less oxygen to swim at a given speed as compared to their initial values. The reasons for this are unknown but could be related to their decreased maintenance metabolic requirements (i.e. reduced SMR), and possibly reduced lipid stores and mass per unit length. In contrast, variables associated with digestive function showed large changes in response to food deprivation. Although total SDA increased when fish were fed after a period of fasting, this was due to a greatly extended SDA duration, especially given that PMR was reduced during this time. This response of depressing digestive function while maintaining locomotor capacity could be an adaptive strategy for reducing energetic costs when food is not available while still remaining primed to avoid predation or to capture food should it appear (Fu et al., 2005; Yan et al., 2015). Indeed, previous work has observed reduced gastrointestinal and liver function during starvation in this species, and these alterations could be partly responsible for the reduction in SMR during fasting (Zeng et al., 2012). It is also possible that digestive duration could increase with efficiency of digestion during periods when food availability is scarce. A combination of an increased SDA duration and a lowered PMR would also act to conserve AS during digestion, allowing for the performance of other physiological functions simultaneously (Norin and Clark, 2017).

### Refeeding restores digestive capacity but diminishes locomotor ability

During refeeding, fish showed an extreme increase in body size, with their average mass increasing by more than 3-fold after 15 days refeeding, a rate much faster than most fish species under comparable conditions (Pang et al., 2016). In general, the southern catfish is a very fast-growing species, with more than 60% of ingested energy being channeled to growth and potentially reaching 3–4 kg in their first year when food is abundant [with maturation occurring at about 5–7 years (Xie and Sun, 1987)]. Accompanying this rapid growth upon re-feeding after food deprivation is an apparent restoration of normal digestive function, with SDA, SDA duration, PMR and PMS all reaching or approaching initial levels.

Several lines of evidence suggest that fish experienced a decline in locomotor performance following 15 days of refeeding after food deprivation. Firstly, *U*_crit_ showed a modest decline with refeeding, despite the large increase in total body length, a metric that would normally be expected to cause an accompanying increase in absolute swimming performance. It is possible that, during refeeding after prolonged food deprivation, individuals experienced a decline in locomotor function due to the negative consequences of compensatory growth (Ali et al., 2003; Killen, 2014; Metcalfe and Monaghan, 2001). This catch-up growth has been noted in other animal taxa, including fish, whereby individuals grow faster than they normally would after a period of reduced growth. Several studies have documented reduced aerobic and anaerobic locomotor performance following compensatory growth (Álvarez and Metcalfe, 2007; Killen et al., 2014; Royle et al., 2006), possibly as a result of compromised muscle structure or other structural elements during rapid tissue formation. It is also possible that the high rate of body mass increase caused an increase in the cost of transport due to increased lipid stores. Additional work, including a parallel treatment in which fish are fed normally throughout the study, is required to specifically examine the role of compensatory growth in the observed decrease in swimming ability and to disentangle the relative contributions of these effects (e.g. structural compromise versus increased mass) in causing a reduction in locomotor ability after refeeding. It is noteworthy, however, that fish that showed the highest growth rates with refeeding showed the greatest reductions in *U*_crit_. The fact that change in body length, in particular, was negatively correlated with the change in *U*_crit_ suggests that changes to structural elements such as bone, cartilage or muscle played a main role in the observed reduction in swimming performance (Nicieza and Álvarez, 2009). It is also possible that changes in energy prioritization and budgeting of available AS may have negatively affected swimming performance during the refeeding period. If refeeding individuals are devoting more AS to growth and digestion, for example, including the maintenance of intestinal tissue, then they may have a reduced aerobic capacity available for swimming activity. This scenario is plausible given that increased rates of feeding and increased growth trajectories can have lingering effects on measures of oxygen uptake even after fish have been allowed to clear their guts before estimation of metabolic rates (Killen, 2014; Rosenfeld et al., 2015). Results of the current study do not support this possibility, however, as specific growth rates (in terms of mass and length gained per day) were not related to either SMR or AS during the refeeding period.

### Food deprivation and refeeding alters trait covariation

Links between SMR and indices related to digestive efficiency seemed particularly sensitive to changes in food availability and feeding. During initial measurements in regularly feeding fish, individuals with a higher SMR also had a lower SDA, SDA duration and PMS, but these correlations were absent in fish that were food deprived and did not re-appear after 15 days of refeeding. The initial links between SMR and metrics of digestive function may be reflective of an increased digestive capacity for individuals with an increased SMR, as these fish digested meals faster and had a higher post-feeding *Ṁ*_O_2__. It is also possible that some individuals had an artificially increased estimate of SMR at the beginning of the study if they exhibited increased stress in response to holding within the respirometry chambers (Burton et al., 2011). An elevated baseline for SMR could lead to an underestimation of SDA in these individuals, contributing to the initially negative correlation between total SDA and SMR. Regardless of the exact causes, shifts in covariation among SMR and indices of digestive function appear to have been mediated by changes in not only the absolute values for SMR but also the degree of among-individual variation in SMR (Killen et al., 2013).

Overall, the flexibility of SMR in response to changing feeding regimes and extent to which variation in baseline function affects relationships among indices of performance suggest that maintenance of energetic requirements is an important component of fitness in this species. The sensitivity of trait covariation to changes in SMR among individuals indicates that correlated selection on traits could occur in some environmental contexts but not others (Kern et al., 2016; Killen et al., 2013; Lande and Arnold, 1983). For example, during fasting, links between SMR and digestive function appear to be uncoupled, and so any selection against increased maintenance metabolism during times of reduced food availability would not also select against digestive function. The observed negative correlation between SMR and AS is unusual compared to other taxa (including fishes), where AS is generally more closely related to MMR with positive links between increased maximum aerobic capacity and maintenance requirements (Auer et al., 2017; Killen et al., 2016b). In the southern catfish, however, increased SMR appears to stem from investment in digestive capacity (e.g. intestinal and liver function) but without an accompanying increase in the MMR, the ‘ceiling’ for overall aerobic capacity. This is possibly due to the ambush predatory lifestyle of this species, which would more likely rely on burst-type anaerobic metabolism for movements during foraging and prey capture (Marras et al., 2010; Marras et al., 2013; Nelson and Claireaux, 2005). If SMR indeed competes with other physiological functions for energy and reduces the available AS for other physiological functions, it is possible that, in general, relatively inactive species may more closely align with the allocation model of energy budgeting as opposed to the production model (Careau et al., 2014, 2008).

### Conclusions

The responses of locomotor and digestive capacity to food deprivation and refeeding showed inverse responses in southern catfish. Fasting caused a depression in digestive function that recovered with refeeding, whereas swimming capacity was robust to fasting but was compromised during resumption of normal feeding. Changes in maintenance metabolism and digestive function with shifting food availability may be beneficial responses that reduce energetic demand when food is scarce (Méndez and Wieser, 1993). Conversely, the reduction in locomotor ability with refeeding after fasting may be a cost of extremely rapid compensatory growth, although this hypothesis requires further examination. Overall, the southern catfish, an exemplar ambush predator, seems to place a high priority on both digestive function and altering maintenance metabolic requirements in accordance with environmental constraints generally while placing a lower priority on locomotor capacity.

## Supplementary Material

Supplementary information
