# Supplementary material for: Digestive and locomotor capacity show opposing responses to changing food availability in an ambush predatory fish
Source: J Exp Biol. 2018 Jun 14;221(12):jeb173187. doi: 10.1242/jeb.173187 (PMC6031316; doi:10.1242/jeb.173187)
Supplement: Supplementary information [file jexbio-221-173187-s1.pdf]

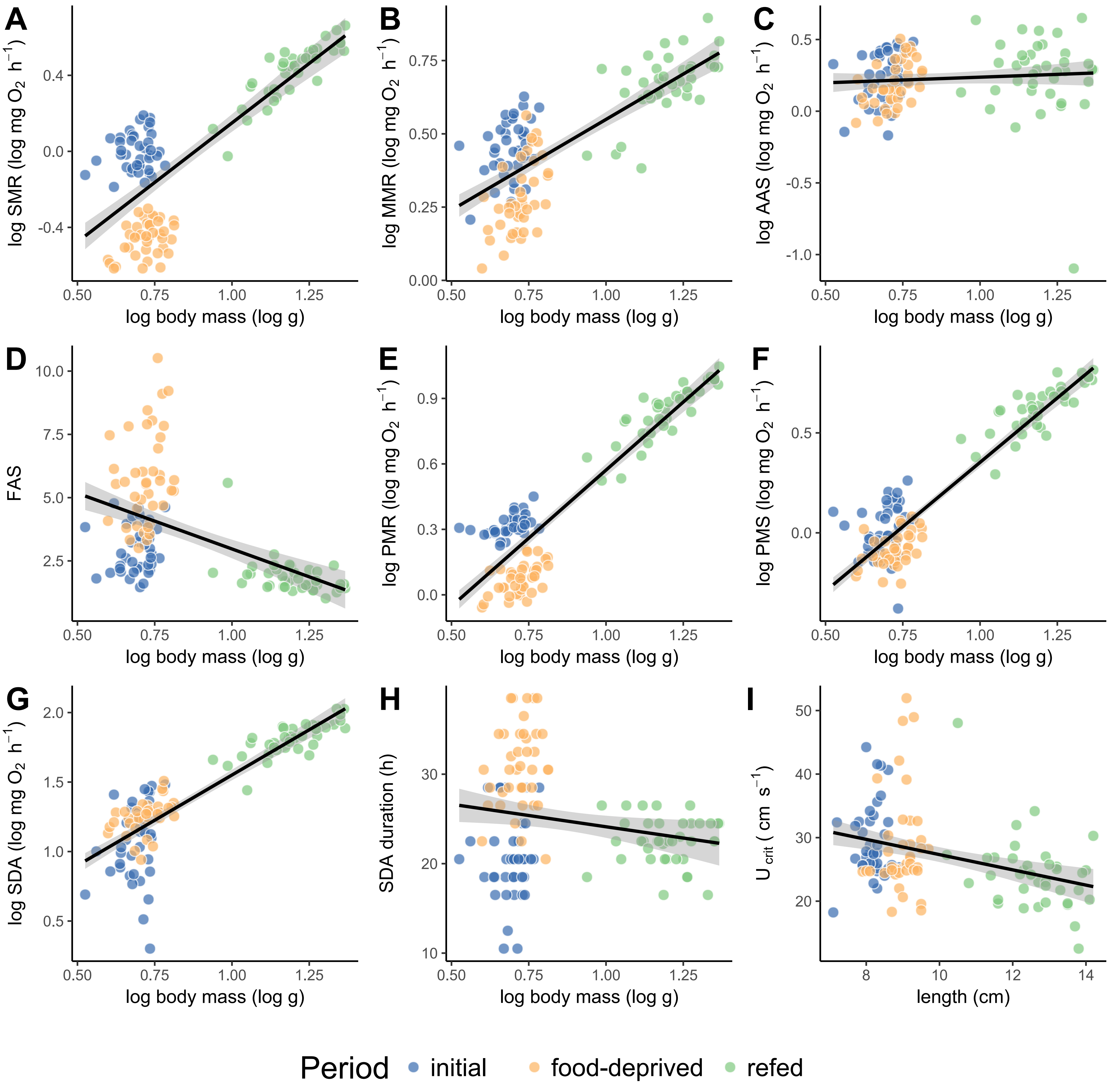

Journal of Experimental Biology • Supplementary information

FIGURE S1. Correlations among metabolic traits and indices of digestive and locomotor function for Southern catfish under conditions of normal feeding (blue), after 15 days of food deprivation (yellow), and 15 days after resuming normal feeding (green). For illustrative purposes, solid lines represent linear regressions; shaded areas around lines represent the 95% confidence intervals. Regression equations are as follows and were used to produce residuals for standardization to a common body mass for visual presentation of data in figures: log SMR = -1.103 + 1.252(log mass), $r^2 = 0.65$, $p < 0.0001$; log MMR = -0.0696 + 0.6187(log mass), $r^2 = 0.60$, $p < 0.0001$; log AAS = 0.159 + 0.0787(log mass), $r^2 = 0.008$, $p = 0.333$; FAS = 7.376 – 4.404(log mass), $r^2 = 0.27$, $p < 0.0001$; log PMR = -0.676 + 1.247(log mass), $r^2 = 0.83$, $p < 0.0001$; log PMS = -0.936 + 1.288(log mass), $r^2 = 0.87$, $p < 0.0001$; log SDA = 0.249 + 1.302(log mass), $r^2 = 0.75$, $p < 0.0001$; SDA duration = 29.162 – 5.039(log mass), $r^2 = 0.04$, $p = 0.029$; U^{crit} = 39.322 – 1.199(length), $r^2 = 0.12$, $p < 0.0001$.

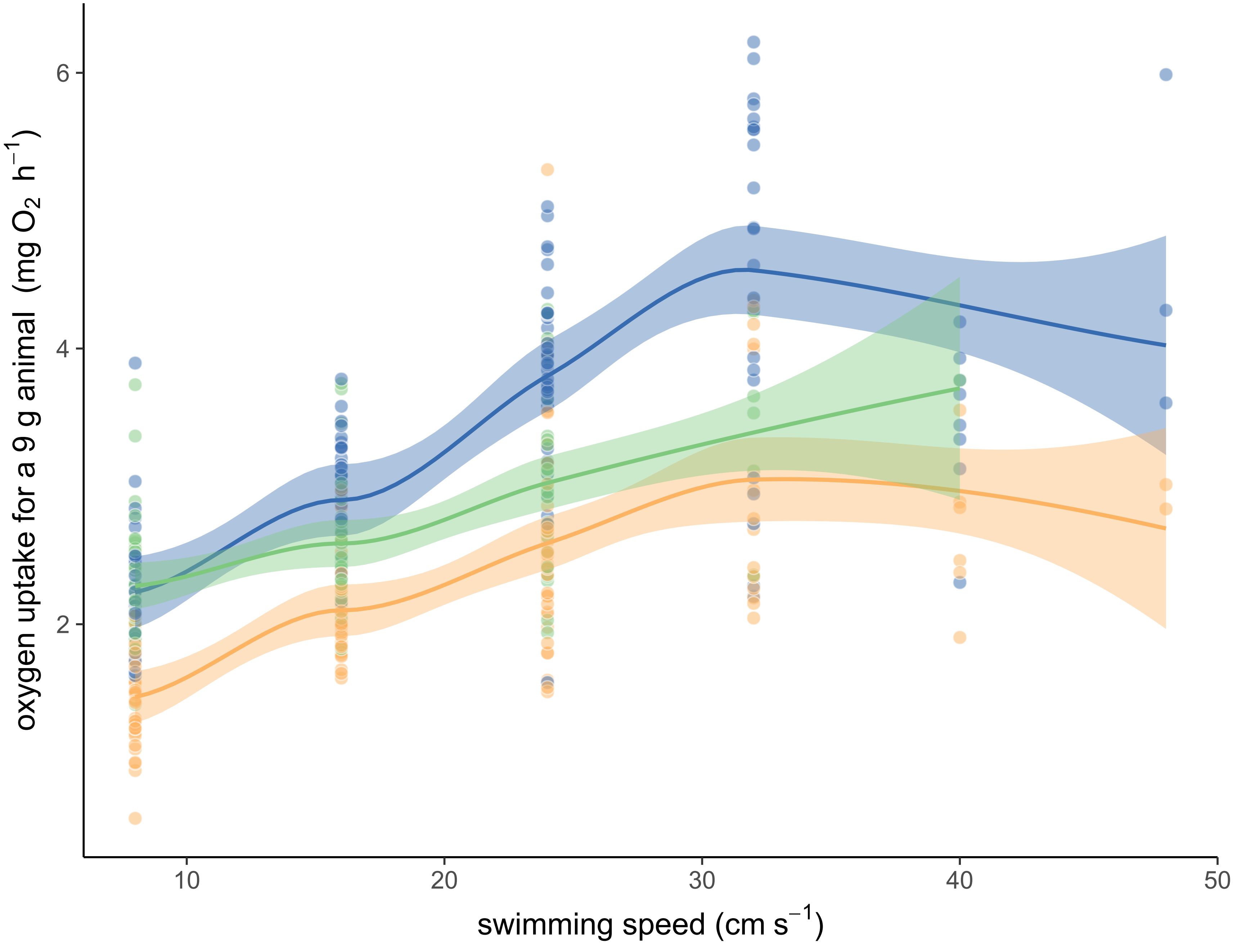
oxygen uptake for a 9 g animal (mg O_2 h^{-1})
6
4
2
10
20
30
40
50
swimming speed (cm s^{-1})

FIGURE S2. Changes in oxygen uptake with swimming speed of Southern catfish under conditions of normal feeding (blue), after 15 days or food deprivation (yellow), and after 15 days of normal feeding (green). Data corresponds to Figure 3 but are expressed here in terms of absolute swimming speed (cm s^{-1}). Each fish was measured under each condition; each data point is data for an individual at a given speed. Rates of oxygen uptake are adjusted to a common body mass of 9 g (the mean mass of all individuals during measurement across the duration of the study). For illustrative purposes, solid lines represent linear regressions; see Table S3 for parameters and results of statistical models. Shaded areas around lines represent the 95% confidence intervals.

Journal of Experimental Biology • Supplementary information

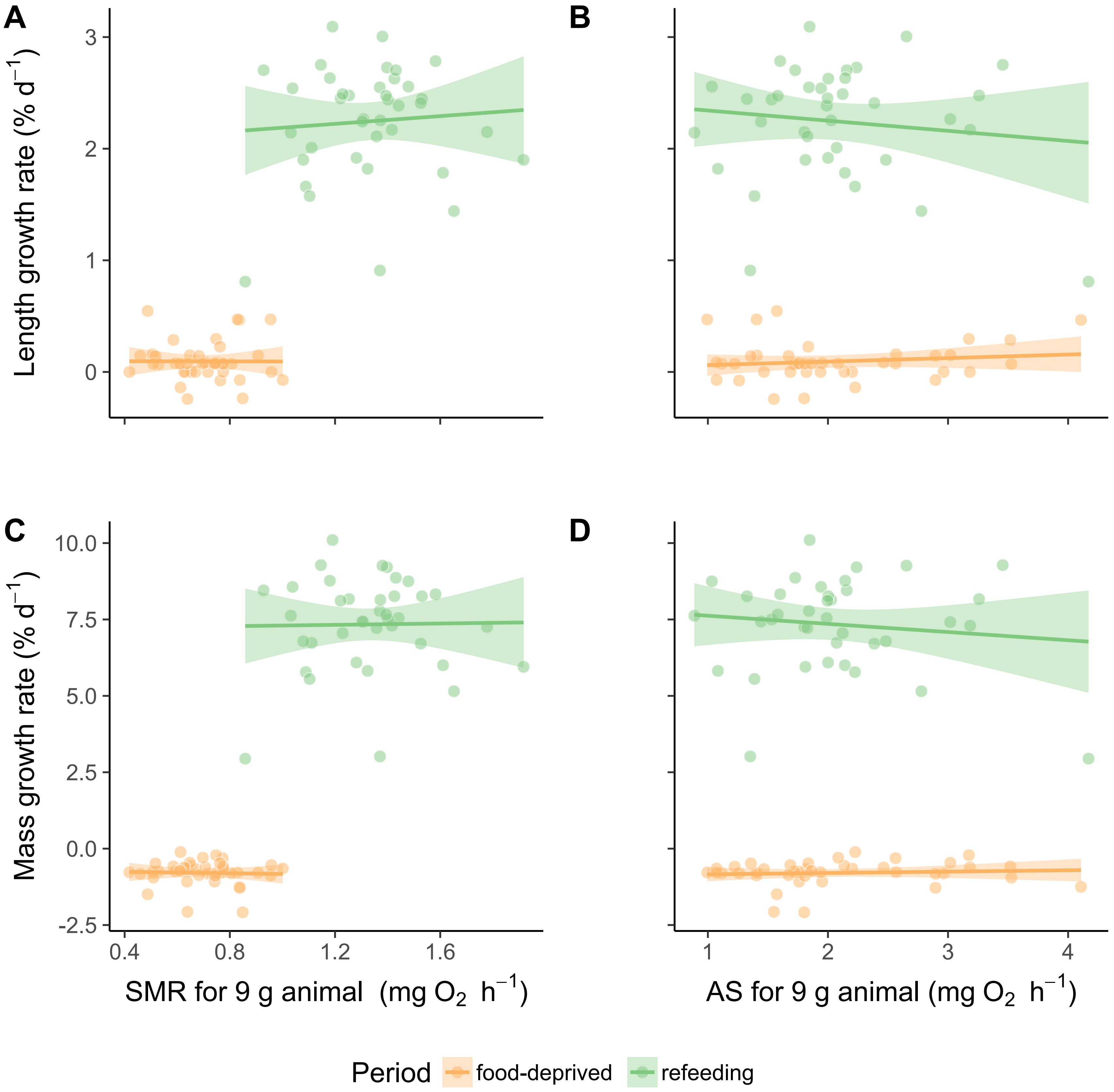
A
B
C
D
Length growth rate (% d^{-1})
Mass growth rate (% d^{-1})
SMR for 9 g animal (mg O_2 h^{-1})
AS for 9 g animal (mg O_2 h^{-1})
Period
food-deprived
refeeding

FIGURE S3. Relationships between growth rate and either standard metabolic rate (SMR) and aerobic scope (AS) in Southern catfish. (A) and (B) depict length specific growth rates; (C) and (D) depict mass specific growth rates. Growth rates were measured during 15 days of food deprivation followed by 15 days of refeeding. Each fish was measured under each condition; each data point is data for an individual during a given feeding period. Rates of oxygen uptake are adjusted to a common body mass of 9 g (the mean mass of all individuals during measurement across the duration of the study). For illustrative purposes, solid lines represent linear regressions; shaded areas around lines represent the 95% confidence intervals.

TABLE S1. Results of linear mixed effects models examining the effects of feeding period on traits associated with morphology and metabolism in Southern catfish (n = 40). Each individual was measured for each response variable three times: (1) initial values at the beginning of the study; (2) after 15 days of food deprivation; and (3) after another 15 days or refeeding (daily to satiation). For feeding period, the reference level is initial values for each response variable. To account for repeated measures, fish ID is included as a random effect in the model.

	estimate	s.e.	df	t	p	r^2_m	r^2_c
**Mass (g)**							
intercept	5.963	0.352	110.53	16.92	< 0.0001	0.832	0.856
feeding period							
fasted	-0.663	0.461	76.69	-1.44	0.154		
refed	10.215	0.468	77.79	21.85	< 0.0001		
**Total Length (cm)**							
intercept	8.928	0.106	86.83	83.93	< 0.0001	0.869	0.923
feeding period							
fasted	0.048	0.116	76.50	0.41	0.682		
refed	3.663	0.117	77.20	31.19	< 0.0001		
**log SMR (log mg O_2 h^{-1})**							
intercept	-0.770	0.091	106.68	-8.50	< 0.0001	0.943	0.953
log mass (log g)	0.991	0.116	106.64	8.56	< 0.0001		
feeding period							
fasted	-0.400	0.019	82.64	-20.79	< 0.0001		
refed	0.006	0.053	111.44	0.12	0.905		

Journal of Experimental Biology • Supplementary information

**log MMR (log mg O_2 h^{-1})**								
intercept		-0.132		0.094	114.00	-1.40	0.164	0.756
0.756								
log mass (log g)	0.75301		0.120	114.00	6.26	< 0.0001		
feeding period								
fasted		-0.128		0.022	114.00	-5.73	< 0.0001	
refed		-0.097		0.055	114.00	-1.74	0.084	
**log AAS (log mg O_2 h^{-1})**								
intercept		0.111	0.215	108.09	0.52		0.606	0.019
0.201								
log mass (log g)	0.168	0.274	108.12	0.61	0.542			
feeding period								
fasted		-0.042		0.045	82.79	-0.94	0.352	
refed		-0.061		0.124	112.15	-0.49	0.626	
**FAS**								
Intercept			3.182		1.273	98.13	2.50	0.014
0.624 0.646								
log mass (log g)	-0.243		1.627	97.58	-0.15	0.882		
feeding period								
fasted	2.753		0.289	83.39	9.54	< 0.0001		
refed		-1.022		0.745	106.50	-1.37	0.173	

SMR: standard metabolic rate (mg O_2 h^{-1}); MMR: maximum metabolic rate (mg O_2 h^{-1}); AAS: absolute aerobic scope (mg O_2 h^{-1}); FAS: factorial aerobic scope

TABLE S2. Results of linear mixed effects models examining the effects of metabolic traits and feeding period on specific dynamic action and indices of aerobic capacity in Southern catfish (n = 40). Each individual was measured for each response variable three times: (1) initial values at the beginning of the study; (2) after 15 days of food deprivation; and (3) after another 15 days or refeeding (daily to satiation). For feeding period, the reference level is initial values for each response variable. To account for repeated measures, fish ID is included as a random effect in the model.

	estimate	s.e.	df	t	p	r^2_m	r^2_c
**SDA (mg O_2)**							
intercept	-33.372	9.529	79.48	-3.50	< 0.0001	0.94	0.94
log mass (log g)	58.696	13.246	89.85	4.43	< 0.0001		
log SMR (log mg O_2 h^{-1})	-71.006	10.767	109.23	-6.60	< 0.0001		
log MMR (log mg O_2 h^{-1})	4.408	6.426	107.18	0.69	0.494		
feeding period							
fasted	0.733	5.530	109.16	0.13	0.895		
refed	5.435	3.957	108.16	1.37	0.172		
log SMR (log mg O_2 h^{-1}) x feeding period							
fasted	54.642	15.901	108.84	3.44	< 0.001		
refed	123.464	14.367	109.60	8.59	< 0.0001		
**SDA duration (h)**							
intercept	18.519	5.684	88.66	3.26	0.001	0.62	0.67
log mass (log g)	1.009	7.832	97.28	0.13	0.898		
log SMR (log mg O_2 h^{-1})	-31.575	6.146	106.24	-5.14	< 0.0001		
log MMR (log mg O_2 h^{-1})	3.223	3.651	103.10	0.88	0.379		
feeding period							
fasted	18.200	3.156	106.28	5.77	< 0.0001		
refed	1.208	2.292	109.67	0.53	0.599		
log SMR (log mg O_2 h^{-1}) x feeding period							

Journal of Experimental Biology • Supplementary information

fasted	47.983	9.066	105.54	5.29	< 0.0001		
refed	30.247	8.278	109.84	3.65	0.0004		
**log PMR (log mg O_2 h^{-1})**							
intercept	0.053	0.066	89.59	0.80	0.425	0.98	0.98
log mass log (g)	0.313	0.091	98.14	3.44	< 0.001		
log SMR (log mg O_2 h^{-1})	0.017	0.079	104.99	0.25	0.807		
log MMR (log mg O_2 h^{-1})	0.053	0.042	101.49	1.26	0.212		
feeding period							
fasted	-0.070	0.036	105.07	-1.91	0.059		
refed	0.136	0.026	109.84	5.12	< 0.0001		
log SMR (log mg O_2 h^{-1}) x period							
fasted	0.319	0.105	104.20	3.05	0.003		
refed	0.552	0.096	109.59	5.77	< 0.0001		
**log PMS (log mg O_2 h^{-1})**							
intercept	-0.444	0.109	82.76	-4.06	0.0001	0.95	0.95
log mass (log g)	0.551	0.151	92.64	3.64	0.0005		
log SMR (log mg O_2 h^{-1})	-1.021	0.121	108.18	-8.42	< 0.0001		
log MMR (log mg O_2 h^{-1})	0.062	0.072	105.54	0.86	0.393		
feeding period							
fasted	-0.037	0.062	108.14	-0.60	0.553		
refed	0.238	0.045	108.89	5.32	< 0.0001		
log SMR (log mg O_2 h^{-1}) x period							
fasted	1.065	0.179	107.64	5.95	< 0.0001		
refed	1.334	0.162	109.96	8.21	< 0.0001		
**log MMR (log mg O_2 h^{-1})**							
intercept	-0.168	0.121	113.00	-1.39	0.168	0.76	0.75
log mass (log g)	0.799	0.155	113.00	5.14	< 0.0001		

log SMR (log mg O_2 h^{-1})	-0.048	0.101	113.00	-0.48	0.634		
feeding period							
fasted	-0.147	0.046	113.00	-3.19	0.002		
refed	-0.096	0.056	113.00	-1.72	0.088		
**log AAS (log mg O_2 h^{-1})**							
intercept	-0.465	0.259	100.97	-1.80	0.075	0.11	0.18
log mass (log g)	0.909	0.331	100.71	2.74	0.007		
log SMR (log mg O_2 h^{-1})	-0.731	0.212	111.46	-3.45	< 0.001		
feeding period							
fasted	-0.334	0.096	112.55	-3.48	< 0.001		
refed	-0.064	0.118	106.17	-0.54	0.590		

SMR: standard metabolic rate (mg O_2 h^{-1}); MMR: maximum metabolic rate (mg O_2 h^{-1}); AAS: absolute aerobic scope (mg O_2 h^{-1}); FAS: factorial aerobic scope; PMR: peak metabolic rate after feeding (mg O_2 h^{-1}); PMS: peak metabolic scope (mg O_2 h^{-1}); SDA: specific dynamic action (mg O_2); SDA duration (h)

Journal of Experimental Biology • Supplementary information

TABLE S3. Results of linear mixed effects models examining the effects of mass and feeding period on log oxygen uptake (log mg O_2 h^{-1}) during swimming in Southern catfish (n = 40). Each individual was measured for each response variable three times: (1) initial values at the beginning of the study; (2) after 15 days of food deprivation; and (3) after another 15 days or refeeding (daily to satiation). For feeding period, the reference level is initial values for each response variable. To account for repeated measures, fish ID is included as a random effect in the model. Two models are presented: the first considers swimming speed relative to fish size (BL s^{-1}) while the second considers speed in absolute terms (cm s^{-1}).

	estimate	s.e.	df	t	p	r^2_m	r^2_c
**Relative Speed**							
intercept	-1.308	0.061	203.60	-21.53	< 0.0001	0.817	0.840
log mass (log g)	0.699	0.084	204.20	8.36	< 0.0001		
speed (BL s^{-1})	0.010	< 0.001	360.90	18.66	< 0.0001		
feeding period							
fasted	-0.201	0.019	342.00	-16.47	< 0.001		
refed	-1.942	0.432	222.20	-4.50	< 0.0001		
**Absolute Speed**							
intercept	-6.134	0.060	200.60	-10.16	< 0.0001	0.720	0.755
log mass (log g)	1.015	0.080	200.90	12.22	< 0.0001		
speed (cm s^{-1})	0.010	< 0.001	361.20	18.66	< 0.0001		
feeding period							
fasted	-0.169	0.011	342.10	-14.20	< 0.001		
refed	-0.168	0.043	219.00	-3.92	< 0.0001		

TABLE S4. Results of linear mixed effects models examining the effects of metabolic traits on critical swimming speed (U_{crit}) in Southern catfish (n = 40), measured in relative (body length s^{-1}) and absolute terms (cm s^{-1}). Each individual was measured for each response variable three times: (1) initial values at the beginning of the study; (2) after 15 days of food deprivation; and (3) after another 15 days or refeeding (daily to satiation). For feeding period, the reference level is initial values for each response variable. To account for repeated measures, fish ID is included as a random effect in the model.

	**estimate**	**s.e.**	**df**	**t**	**p**	r^2_m	r^2_c
**U_{crit} (BL s^{-1})**							
intercept	6.612	0.887	111.98	7.46	< 0.0001	0.21	0.48
log mass (log g)	-5.268	1.221	110.31	-4.32	< 0.0001		
log SMR (log mg O_2 h^{-1})	-0.139	0.698	106.35	-0.20	0.843		
log MMR (log mg O_2 h^{-1})	2.338	0.617	91.27	3.9	0.0003		
feeding period							
fasted	-0.341	0.320	96.24	-1.07	0.288		
refed	0.161	0.403	111.58	0.40	0.689		
**U_{crit} (cm s^{-1})**							
intercept	47.178	8.153	111.94	5.786	< 0.0001	0.21	0.48
log mass (log g)	-35.487	11.217	110.00	-3.164	< 0.002		
log SMR (log mg O_2 h^{-1})	-2.775	6.417	105.78	-0.432	0.666		
log MMR (log mg O_2 h^{-1})	21.281	5.657	90.47	3.762	0.0003		
feeding period							
fasted	-0.217	2.932	95.51	-0.074	0.941		
refed	6.644	3.703	111.41	1.794	0.076		

SMR: standard metabolic rate (mg O_2 h^{-1}); MMR: maximum metabolic rate (mg O_2 h^{-1})

TABLE S5. Results of general linear models examining the effects of growth rate on the percentage change in critical swimming speed (body lengths per second; U_{crit}) after the 15 day recovery period after food-deprivation mass in Southern catfish (n = 40).

	estimate	s.e.	df	t	p	r^2
**All data - mass**						
intercept	61.410	19.501		3.149	0.0033	0.27
mass growth (% d^{-1})	-9.581	2.601	36	-3.684	0.0008	
**All data - length**						
intercept	50.204	19.065		2.633	0.0124	0.21
length growth (% d^{-1})	-26.325	8.289	36	-3.176	0.0031	
**Outlier removed - mass**						
intercept	31.096	20.070		1.549	0.1303	0.12
mass growth (% d^{-1})	-5.732	2.647	35	-2.166	0.0372	
**Outlier removed - length**						
intercept	19.327	19.463		0.993	0.328	0.07
length growth (% d^{-1})	-13.563	8.363	35	-1.622	0.114	
